# Mini-invasive treatment of a large pseudoaneurysm of the neck related to central venous catheter placement: A case report

**DOI:** 10.1097/MD.0000000000011262

**Published:** 2018-07-20

**Authors:** Chiara Palermo, Angelo Sanfiorenzo, Alessia Testo Giaquinta, Carla Virgilo, Massimiliano Veroux, Pierfrancesco Veroux

**Affiliations:** Vascular Surgery and Organ Transplant Unit, Azienda Ospedaliero-Universitaria Policlinico, Catania, Italy.

**Keywords:** central venous catheter, complications, endovascular repair, pseudoaneurysm, stent-graft, thyro-cervical trunk, thyroid artery

## Abstract

**Rationale:**

Central venous catheter (CVC) placement, particularly in emergency setting, may be associated with significant morbidity and mortality.

**Patient concerns:**

A 33-year old woman with suspected pulmonary embolism, developed a pseudoaneurysm of the neck three days after a CVC placement in the right internal jugular vein, determining compression to adjacent neck structures.

**Diagnoses:**

Computed tomography angiography and selective angiography demonstrated the presence of the pseudoaneurysm originating from the thyro-cervical trunk.

**Interventions:**

The treatment was minimally invasive with endovascular exclusion first, and an open thrombectomy to resolve compressive syndrome two days later.

**Outcomes:**

The color Doppler ultrasound confirmed the complete exclusion of the pseudoaneurysm with patency of the thyroid artery. A comprehensive review of literature on the risk factors and management of the unintended artery puncture was included.

**Lessons:**

A correct technique under ultrasound guidance may reduce the incidence of unintended arterial injury during CVC placement. In patients with suitable anatomy and unfit for open repair, a minimally invasive approach provides a safe alternative to open surgery with excellent results.

## 1 Introduction

Central venous catheters (CVC) are indwelling devices for daily clinical practice for treatment of several diseases.^[1]^ The internal jugular vein (IJV) is the preferred site for CVC placement and appropriate venous puncture under real-time ultrasound guidance usually provides an easy access to venous system even in an emergency condition. However, CVC placement may be associated with severe and potentially life-threatening complications. Mechanical complications are the most frequent and they are usually immediate and associated with a significant mortality rate.^[2]^ In addition, these complications may increase the length and costs of hospital stay as well as the need for subsequent interventions.^[2]^

The most common mechanical complication associated with IJV cannulation is accidental arterial puncture, mainly the carotid artery, due to its close proximity to the IJV, but also the vertebral artery,^[3]^ the thyrocervical trunk,^[4]^ innominate artery,^[5]^ and the internal mammary artery,^[6]^ with an incidence of 4.2% to 9.3%.^[7]^

While the accidental arterial puncture is mostly harmless, and treated solely with an adequate local compression, an inadvertent insertion of a CVC in a cervicothoracic artery may have serious consequences, such as compression of the airways, pseudoaneurysm, hemothorax, arteriovenous fistula, stroke, massive bleeding, and death.^[8]^

We present a unique case of endovascular repair of a right thyro-cervical trunk pseudoaneurysm, developed after a placement of a CVC in the right IJV. The patient involved in this study gave her written informed consent authorizing all procedures and provided informed consent for publication of the case, and ethical consent for this study was not necessary as it did not involve any experimental treatment, and does not reveal any confidential information about patient that could violate her privacy.

A comprehensive review of literature on the risk factors and management of the unintended artery puncture was included.

## 2 Case report

A 33-year-old woman was admitted to the intensive care unit after emergency cesarean section for respiratory failure due to suspected pulmonary embolism. The patient had clinical history of heterologous artificial insemination, without any evidence of deep vein thrombosis, or history of coagulopathy.

A CVC was placed in the right IJV for intravenous infusion of inotropes and sodium heparin under real time ultrasound guidance. During the procedure, there was no sign of accidental arterial puncture and no swelling was observed at the end of CVC placement.

**Figure 1:**
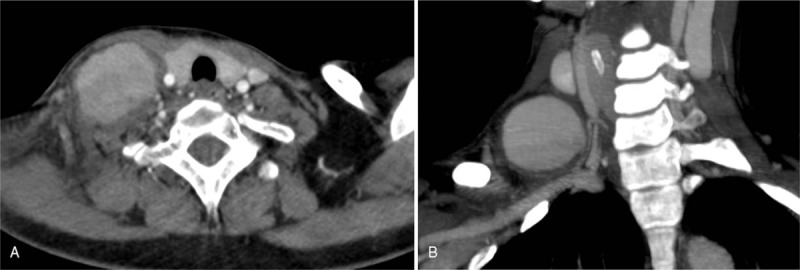
Cervical computed tomography angiography in axial (A) and sagittal (B) view, showing a large false aneurysm of the neck, with a likely origin from the thyrocervical trunk, with no signs of peri-arterial bleeding or hematoma.

**Figure 2:**
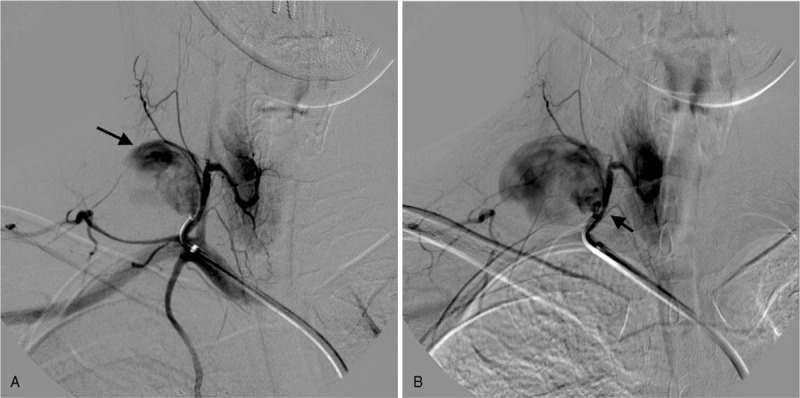
Selective Angiography of the right thyro-cervical trunk confirmed the presence of the pseudoaneurysm (A, arrow) with active communication with the artery (B, arrow).

**Figure 3:**
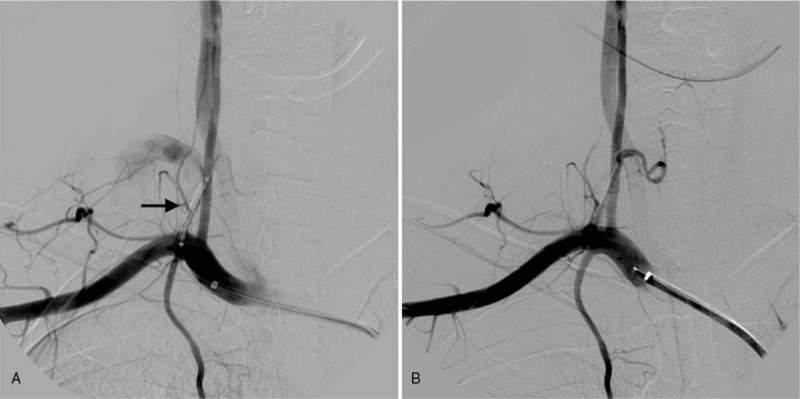
Endovascular repair of the pseudoaneurysm. A balloon expandable covered stent was released in the thyro-cervical trunk (A, arrow), and the post-procedure angiography confirmed the complete exclusion of the pseudoaneurysm and the patency of the inferior thyroid artery (B).

A thoraco-abdominal Computed Tomography (CT) angiography was then performed, showing a subtotal atelectasis of the left lung with the presence of a pulmonary arterio-venous malformation. The patient underwent the embolization of pulmonary arterio-venous malformation and uterine arteries. However, due to massive hemothorax, the patient underwent an urgent left thoracotomy. In the early postoperative period, 3 days after the CVC placement, the patient complained dysphonia, swallowing disorder with progressive breathing insufficiency. A physical examination revealed the presence of a pulsatile mass in right lateral cervical region. A CT angiography of the neck demonstrated the presence of a 45-mm pseudoaneurysm in the right cervical region, compressing the IJV and adjacent neck structures (Fig. 1). The patient was considered at risk for open repair due to high risk of intraoperative bleeding and for concomitant comorbidities, and a 2 steps procedure was planned. The first procedure was performed through a retrograde right common femoral artery access under sonography guidance. After 6-Fr, 55-cm-long sheath introduction (Flexor RAABE, Cook Medical Inc., Bloomington, IN), 5000 units of heparin were administered, and a BER II diagnostic catheter (4-Fr, 100-cm, Cordis Corporation, Bridgewater, NJ) was advanced into the brachiocephalic trunk with the support of a 0,035” hydrophilic guidewire (Cordis Aquatrack; Baar, Switzerland). In coaxial the sheath introducer was placed in right subclavian artery and the BER II was advanced till the origin of thyro-cervical trunk. A preliminary angiography demonstrated the presence of a giant false aneurysm of the right thyro-cervical trunk with its entry tear located below the right ascending cervical artery (Fig. 2). Due to the large size of the sac, coil embolization was not preferred, and a balloon-expandable covered stent (Advanta V12, Atrium Medical Corporation, Merrimack, NH) was introduced over a 0,014” guidewire (Hi-Torque Command, Abbott Vascular, Santa Clara, CA) and deployed in the right thyro-cervical trunk achieving the complete coverage of communication between artery and pseudo aneurysm sac (Fig. 3).

Two days after the endovascular repair, a color-doppler ultrasound of the neck confirmed the absence of flow and the complete thrombosis of the pseudoaneurysm sac, which was finally surgically drained in local anesthesia to resolve the compressive symptoms.

## 3 Discussion

To our knowledge, this is the first reported case of thyro-cervical trunk pseudoaneurysm developed after CVC placement in the IJV. The management or consequence of arterial punctures is directly related to the size of needle or catheter during CVC placement. The definition of the large-bore catheter is mentioned in some literatures, which indicate unintended arterial cannulation with a 7 French or larger catheter or dilator.^[7,9,10]^ While accidental arterial puncture caused by smaller needle (20-G to 25-G) is usually without clinical consequences, arterial injuries with large-bore catheters may determine devastating complications on carotid or vertebral arteries.^[9]^ Anatomical variations, obesity, history of neck surgery and vessel cannulation, low-experience operator, extreme rotation of the neck, multiple needle attempts, large-bore needle, left-side cannulation, too deep (>2 cm) or too laterally needle insertion, coagulopathy, are all reported as risk factors for iatrogenic arterial puncture.^[3,9,11]^ Standard access using anatomic landmarks has been recently increasingly replaced by the ultrasound-guided approach: meta-analyses of randomized controlled trials^[12–16]^ indicate that, compared with the landmark-guided technique, real-time ultrasound guided venipuncture of the internal jugular vein has a higher first insertion attempt success rate, reduced access time, higher overall successful cannulation rate, and decreased rates of arterial puncture.

Pseudoaneurysms of thyroid artery are extremely rare, and only 3 case reports are described in literature.^[17–19]^ Debek et al^[17]^ reported a patient with platelet abnormality, who developed an inferior thyroid artery injury with focal false aneurysm after multiple attempts of CVC placement with 22 gauge finder needle. The false aneurysm of inferior thyroid artery was successfully embolized with coils. Schummer et al^[18]^ described a 71-year old patient in whom an unintended inferior thyroid artery cannulation during CVC placement was surgically treated. More recently, Ruan et al^[19]^ described an iatrogenic inferior thyroid artery pseudoaneurysm during unsuccessful central line placement treated with micro-coil embolization.

**Figure 4:**
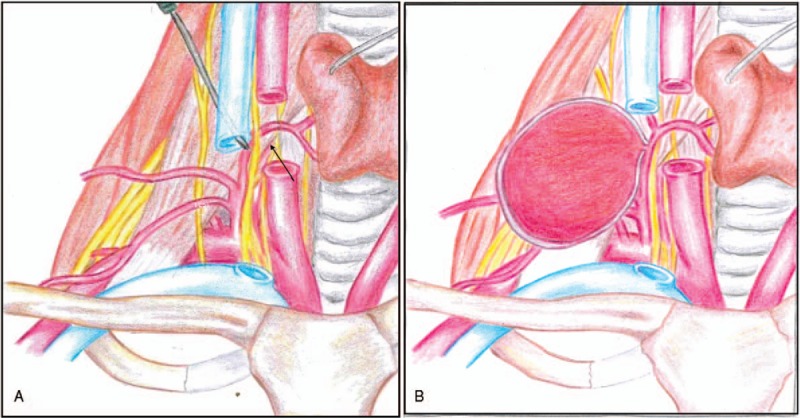
Mechanism of arterial injury. During the central venous catheter placement the needle was pushed too deep in the neck causing an accidental puncture of the thyro-cervical trunk (A, arrow). The unrecognized arterial injury in a patient with anticoagulation therapy slowly progressed to the formation of a giant pseudoaneurysm (B).

In the present case, we attributed the formation of the thyro-cervical trunk pseudoaneurysm to the following factors: the introducer needle was inserted too low and deep in the neck, crossing the lumen of the IJV, with subsequent puncture of the thyro-cervical trunk (Fig. 4); the CVC placement was completed correctly, but the physician was not aware of the arterial injury, so that the arterial puncture was not compressed; the presence of anticoagulation therapy delayed the closure of the arterial injury, which slowly progressed to the formation of the pseudoaneurysm (Fig. 4).

Prevention strategy includes the real-time ultrasound guided venipuncture, and arterial puncture should be suspected in case of difficult progression of the CVC or in case of sudden pulsatile mass in the cervical region. Physical examination, CT-angiography and color-doppler ultrasound could be helpful to confirm the diagnosis.

The management of arterial injury during IJV catheterization may be challenging. Open surgery, presents technical difficulties and a high morbidity rate,^[20,21]^ while endovascular techniques provide a mini-invasive and safe treatment of such complications.^[17,19]^ Endovascular techniques using covered stent grafts may provide a technically simple, safe, and durable solution to supra-aortic vascular injuries,^[21–23]^ and the low-rate of complications favor this treatment for high risk patients, despite covered stents long term durability and safety remain unknown because of limited number of reports.^[24]^ While endovascular exclusion of pseudoaneurysm with microcoils has been described,^[17,19]^ in this young patient the use of microcoils, while promoting the rapid pseudoaneurysm thrombosis, did not resolve the compressive symptoms and the artery embolization would permanently close the vessel and its distal branches. Indeed, the covered stent allowed for complete pseudoaneurysm exclusion while maintaining the inferior thyroid artery patency.

These stent-grafts take the advantages of a precise positioning without migration during release of the stent and the graft may be tailored to the vessel caliber by overdilating the graft, to reduce the risk of endoleak:^[24]^ this resulted in a good safety and efficacy profile when used for the treatment of traumatic extracranial internal carotid artery injury.^[25]^

The management of consequence of arterial placement of a large-bore catheter may be challenging. As soon as the operator suspects a large-bore catheter in artery, he should leave the catheter in place while planning the correct treatment. Percutaneous vascular closure devices (VCD) have been usually used in interventional cardiology to obtain a rapid and efficient hemostasis of femoral access after coronary and endovascular interventions.^[26]^ Only recently, the use of different types of VCD has extended from the femoral artery to other vascular fields (eg, carotid, subclavian, and popliteal) and thanks to their associated simplicity of use and efficiency in obtaining hemostasis, VCDs have become a real alternative to open surgery in treating incidental arterial CVCs placement.^[27–30]^ The choice of one VCD instead of another must be individualized according to local factors (eg, vessel anatomy, tortuosity, presence of calcifications, plaques, and bifurcations) and most importantly according to the operator's knowledge of the device's characteristics. Schutz et al^[31]^ described the case of a 79-year-old patient presenting with cardiogenic shock due to erroneously placed CVC in the right subclavian artery, in whom the hemostasis was obtained with the insertion of a 8 Fr Angioseal TM closure device. Alternative treatment for unintended puncture of small arteries such as internal mammary artery and inferior thyroid artery is its selective embolization^[8,17,19]^: Chemelli et al^[6]^ reported 5 patients with internal mammary artery bleeding after subclavian vein puncture who were successfully treated through embolization with microcoils, with no procedure-related complications.

In conclusion, we reported the first iatrogenic pseudoaneurysm of thyro-cervical trunk during CVC placement in IJV. A 2-step procedure was used to maintain the artery patency, cover the communication, and resolve the compressive symptoms caused by the pseudo-aneurysm. In patients with suitable anatomy, a mini-invasive approach may provide an immediate excellent result, although long-term patency remains unknown because of limited number of reports.

## Author contributions

**Conceptualization:** Alessia Testo Giaquinta, Carla Virgilio, Pierfrancesco Veroux.

**Data curation:** Chiara Palermo, Angelo Sanfiorenzo, Alessia Testo Giaquinta, Carla Virgilio, Massimiliano Veroux, Pierfrancesco Veroux.

**Investigation:** Chiara Palermo, Angelo Sanfiorenzo, Alessia Testo Giaquinta, Carla Virgilio, Massimiliano Veroux, Pierfrancesco Veroux.

**Methodology:** Alessia Testo Giaquinta, Carla Virgilio, Massimiliano Veroux, Pierfrancesco Veroux.

**Supervision:** Massimiliano Veroux, Pierfrancesco Veroux.

**Validation:** Pierfrancesco Veroux.

**Writing – original draft:** Chiara Palermo, Angelo Sanfiorenzo.

**Writing – review & editing:** Massimiliano Veroux, Pierfrancesco Veroux.
